# Impact of laparoscopic ultrasound during PIPAC directed treatment of unresectable peritoneal metastasis

**DOI:** 10.1515/pp-2024-0007

**Published:** 2024-09-06

**Authors:** Magnus S. Jørgensen, Alan P. Ainsworth, Claus W. Fristrup, Michael B. Mortensen, Martin Graversen

**Affiliations:** Odense PIPAC Center, Department of Surgery, 11286 Odense University Hospital, Odense, Denmark

**Keywords:** clinical impact, laparoscopic ultrasound, liver metastases, peritoneal metastasis, pressurized intraperitoneal aerosol chemotherapy

## Abstract

**Objectives:**

Laparoscopic ultrasound (LUS) combines both laparoscopy and ultrasound imaging of the peritoneum liver and retroperitoneum. LUS has not been described in treatments with pressurized intraperitoneal aerosol chemotherapy (PIPAC). We present our experience with LUS in patients undergoing PIPAC.

**Methods:**

Retrospective study of LUS findings from the prospective PIPAC-OPC2 trial. Main outcome was changes in overall treatment strategy due to LUS findings.

**Results:**

PIPAC-OPC2 included 143 patients of which 33 patients were treated with electrostatic precipitation PIPAC. Nine patients were excluded due to primary non-access. During PIPAC 1, LUS was performed in 112 of 134 (84 %) PIPAC procedures and changed overall treatment strategy in one patient due to detection of multiple liver metastases unseen by baseline CT. During PIPAC 2 and 3 LUS was performed in 59 of 104 (57 %) and 42 of 78 (54 %) PIPAC procedures, respectively. Throughout PIPAC 1–3, LUS also detected pathological lymph nodes in 16 patients, and focal liver lesions in another four patients of uncertain origin. No further examinations were performed in these patients, and the overall treatment strategy was not changed according to the PIPAC-OPC2 protocol. One patient had a splenic capsule rupture related to the LUS itself. This was managed conservatively.

**Conclusions:**

LUS may be safely performed during PIPAC. However, LUS has limited clinical impact in patients scheduled for PIPAC, and cannot be recommended as a routine procedure when performing PIPAC.

## Introduction

Peritoneal metastasis (PM) is often seen in the late stage of many solid tumors. The prognosis is poor and patients are faced with poor quality of life due to ascites, bowel obstruction and general fatigue. Systemic chemotherapy has a short and limited effect on PM, and pressurized intraperitoneal aerosol chemotherapy (PIPAC) was introduced as a local treatment option a decade ago 1], [2], [3], [4. PIPAC provides significant and measurable local effect on PM with small impact of patients’ quality of life 5], [6], [7], [8. PIPAC, which is normally provided in series of three procedures, is safe, well tolerated and may be repeated until disease progression [9]. However, PIPAC only treats PM and not extraperitoneal metastasis in the liver and/or retroperitoneum. Some patients with limited and stable extraperitoneal metastasis are receiving both systemic chemotherapy and PIPAC (bidirectional treatment), but more often extraperitoneal metastases are considered exclusion criteria to PIPAC [10].

Computed tomography (CT) scan, positron emission tomography-CT (PET-CT) and magnetic resonance imaging (MRI) are used to diagnose PM [11]. However, these imaging modalities have limitations regarding the diagnosis and extent of PM as well as evaluating treatment response 11], [12], [13. In addition, the assessment of small-volume extraperitoneal malignant disease which might exclude the patient from receiving PIPAC, may also be difficult. For the diagnosis of liver metastases, CT has shown a sensitivity of 75 % in patients with gastric cancer as primary tumor and 87 % for patients with liver metastases from colo-rectal cancer, whereas PET-CT showed a sensitivity for detecting liver metastases of 70 % in patients with gastric cancers and 96 % in patients with colo-rectal cancers [14, 15]. The sensitivity of MRI in detection of liver metastasis among patients with colo-rectal cancer is reported as 87 % in a per-patient based analysis, while being 89 % in a per-lesion based analysis [14, 16]. Test accuracy for CT and PET/CT in detecting para-aortic and pelvic lymph node metastases among patients with ovarian cancers has also been studied and shown sensitivities of 47 % and 81 %, respectively [17].

**Figure 1:**
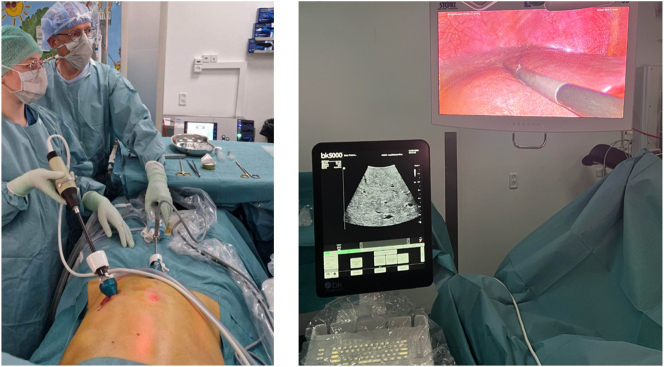
Left: the laparoscopic ultrasound (LUS) probe is inserted through the 12 mm trocar, and the camera through the 5 mm trocar. Right: set-up with the ultrasound screen to the left and the laparoscopic screen to the right.

Laparoscopic ultrasound (LUS) combines laparoscopy and ultrasound providing detailed imaging of intraperitoneal structures including liver parenchyma and retroperitoneum in addition to the peritoneum. LUS can be used as a supplement to standard imaging procedures for staging patients with intraabdominal malignant diseases (Figure 1). It is easy and safe to perform, and it may add clinically significant information to the pretherapeutic staging procedures 18], [19], [20], [21.

In order to try to ensure that extraperitoneal cancer spread was not missed during both first and additional PIPAC procedures in patients with PM, we introduced LUS as part of the PIPAC procedure in 2016. Since LUS has not been included and evaluated in relation to the PIPAC procedure, we decided to study the potential clinical effect of LUS during the initial and subsequent PIPAC procedures in patients with PM from various primary tumors. The study rationale being that we would be able to detect extra-peritoneal metastasis not seen by conventional imaging, and by this avoid futile PIPACs in patients with already too widespread disseminated disease. Thus, the aim of this present study was to see whether LUS revealed findings otherwise not detected by the standard pretherapeutic evaluation and the potential clinical impact of such findings.

## Materials and methods

Retrospective analysis of the findings at LUS performed in patients who were included in a large, prospective phase II trial evaluating the effect of PIPAC directed therapy in patients with PM (PIPAC-OPC2 trial). Details regarding this study have been published previously [6]. In short, patients with PM from abdominal cancers, a maximum of one extraperitoneal metastasis, and in performance status 0–1 were treated with PIPAC. Treatment was planned in series of three PIPACs. Pretherapeutic evaluation of patients was made by a contrast-enhanced CT scan of the chest and abdomen performed within one month prior to PIPAC 1. All patients had a follow-up CT scan two weeks after PIPAC 3, and the findings of this was discussed at the multidisciplinary tumor board conference regarding additional PIPAC treatment. The present study’s only exclusion criteria was non-access during PIPAC 1.

### LUS

All surgeons were certified in PIPAC and experienced in LUS. The LUS procedure was completed as part of each staging laparoscopy before nebulization of intraperitoneal chemotherapy. The LUS probe (9066 4-Way laparoscopic transducer, connected to the ultrasound screen (bk3000-01, BK Medical, Copenhagen, Denmark)) was introduced through the 12 mm trocar that was later used for the nebulizer. The liver and retroperitoneum were scanned to detect liver- and/or lymph node metastases. The LUS procedure was considered complete when both liver lobes and the retroperitoneum had been evaluated. In case the LUS findings could change the overall treatment strategy, a short conference with another surgical oncologist was made in the operating room.

### Outcomes

The main outcome of this study was to investigate if LUS before PIPAC 1 changed the overall treatment strategy. A change of treatment strategy was defined as either a cancellation of PIPAC, or added systemic chemotherapy due to extraperitoneal findings. Secondary, we investigated if LUS before PIPAC 2 or 3 changed overall treatment strategy.

### Data collection

Data were collected from the database used for the PIPAC-OPC2 study. We collected prospective data on time used for LUS, LUS findings, agreement with baseline CT, and if the findings led to change of overall treatment strategy. Additional information regarding findings and completeness of LUS, CT, or clinically relevant information were found in patient files.

### Ethics

This study is a retrospective analysis of patients who consented to the prospective PIPAC-OPC2 study [6].

## Results

**Figure 2:**
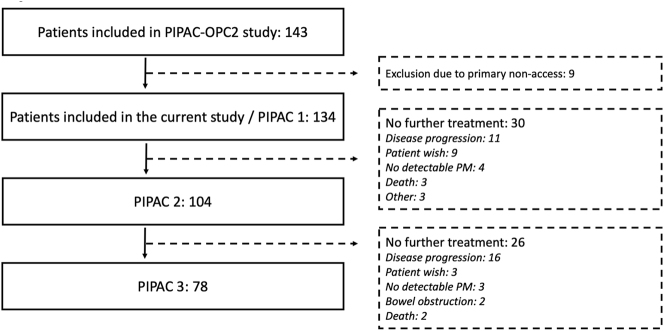
Patient flow from PIPAC 1 to PIPAC 3.

The PIPAC-OPC2 study included 143 patients, from December 2016 to January 2022. Thirty-three of these received electrostatic precipitation PIPAC. Of the 143 patients, nine were excluded due to primary non-access to the abdominal cavity (Figure 2). Demographic data, primary tumor origin and previous treatments regarding the 134 patients included in the final analysis, are listed in Table 1.

**Table 1:** Demographic data, primary tumor origin and previous treatments.

Basic characteristics	Total
Number of patients	134
Age, median years (range)	62 (31–80)
Gender, male (%)	52 (39)
Performance status	
ECOG, 0 (%)	57 (43)
ECOG, 1 (%)	77 (57)
Primary tumor	
Stomach	35
Colon/rectum	28
Pancreas	23
Ovaries	19
Appendix	10
Bile duct	5
Small bowel	5
Esophagus	4
Malignant peritoneal mesothelioma	2
Breast	1
Cervix uteri	1
Metastasis of unknown primary	1
Resection of primary tumor performed (%)	63 (47)
Previous oncological treatment	
Systemic palliative chemotherapy (%)	131 (98)
CRS and HIPEC	5 (4)
Bidirectional treatment (%)	56 (42)
Extraperitoneal metastasis at inclusion (%)	23 (18)
Abdominal wall	6
Lung	4
Liver	3
Pleura	2
Mediastinum	2
Local relapse in bowel anastomosis	2
Ovaries	2
Retroperitoneum	1
Cervix uteri	1

CRS, cytoreductive surgery; ECOG, Eastern Cooperative Oncology Group; HIPEC, hyperthermic intraperitoneal chemotherapy.

During PIPAC 1, LUS was performed in 112 of 134 (84 %) PIPAC procedures and LUS was complete in 83 of the procedures (75 %) (Table 2). The median time spent on LUS during PIPAC 1 was 5 min (range 1–14).

**Table 2:** Findings at laparoscopic ultrasound (LUS).

	PIPAC 1, n (%)	PIPAC 2, n (%)	PIPAC 3, n (%)
No. of patients	134	104	78
LUS performed			
Yes	112 (84)	59 (57)	42 (54)
No	22 (16)	45 (43)	36 (46)
Reasons			
Adhesions	13	5	7
Not considered routine	0	38	25
Not specified	9	2	4
LUS complete			
Yes	83 (75)	39 (66)	24 (57)
No	29 (25)	20 (34)	18 (43)
LUS changed overall treatment strategy			
Yes	1 (1)	0 (0)	0 (0)

During PIPAC 2 and 3, LUS was performed in 59 of 104 (57 %) and 42 of 78 (54 %) procedures, respectively. LUS was complete in 39 (66 %) and 24 (57 %) of these, and the median time spent on LUS was 4 min (range 1–10).

In one procedure (0.5 %), the LUS probe caused injury of the splenic capsule, which was managed conservatively. The PIPAC treatment continued after 15 min of hemostasis, and the postoperative recovery was uneventful. No other intraoperative complications related to the LUS procedure were seen.

### Findings at LUS changing the therapeutic strategy

LUS found multiple liver metastasis in one patient during PIPAC 1, which had not been detected by baseline CT (Table 2). This led to a cancellation of the planned PIPAC. In another patient, LUS showed multiple liver metastasis at PIPAC 3, which also led to a cancellation of the PIPAC treatment. However, in this patient the liver metastases were also observed during laparoscopy and not only by LUS.

### Findings at LUS not changing therapeutic strategy

At PIPAC 1, LUS found a pathological retroperitoneal lymph node (defined as enlarged, round, and homogeneous in structure) in 11 patients which had not been detected by baseline CT. No biopsies were taken.

Liver metastases were suspected due to findings at LUS in three patients during PIPAC 1. No biopsies were taken, and the suspicious lesions were also observed by LUS during PIPAC 2 and PIPAC 3. They remained unchanged or disappeared, and no liver metastases were detected during LUS in the following PIPACs in those three patients.

During PIPAC 2, LUS found two patients with pathological retroperitoneal lymph nodes not detected at baseline CT or at PIPAC 1.

During PIPAC 3, LUS found a suspicious liver lesion in one patient. At follow-up CT there were no visible lesions thus it was concluded that the patient had no metastases in the liver. LUS also detected pathological lymph nodes in retroperitoneum in three patients during PIPAC 3 which had not been detected at previous LUS or baseline CT.

The median time from baseline CT to new findings detected at LUS during PIPAC 1 was 14 days (range 1–54).

## Discussion

No previous study has investigated the potential clinical impact of LUS in patients with PM scheduled for PIPAC. Our data showed that LUS could be safely performed during PIPAC, and that LUS detected additional liver lesions and/or retroperitoneal lymph nodes in 22 patients throughout PIPAC 1–3. However, only in one patient did these findings change the overall treatment strategy due to detection of multiple liver metastases prior to PIPAC 1.

LUS also found liver metastases in one patient during PIPAC 3, but these were already detected at laparoscopy. Thus, the supplement of LUS was not considered to change the overall treatment strategy.

LUS detected previously unseen pathological lymph nodes in retroperitoneum in 16 patients (12 %) throughout PIPAC 1–3. LUS gave rise to suspicion of liver metastases in four patients (3 %). None of the findings initiated further examinations or changed the overall treatment strategy because these findings were not considered relevant to alter treatment strategy according to the PIPAC-OPC2 protocol. However, if these findings would have changed the treatment strategy, the clinical impact of LUS would have been higher.

Similar results about LUS suspected liver metastases were observed in a randomized study on patients with colo-rectal cancer. This study found four patients where LUS suspected liver metastases, but it turned out to be benign liver lesions (14). None of the surgeons were blinded, and performance bias might had occurred. Thus, the study concluded that LUS during laparoscopic surgery for primary colorectal cancer could not be recommended.

In patients with gastric, pancreatic, and colo-rectal cancer scheduled for curative surgery, the addition of LUS changed the therapeutic strategy in 2 % [20, 22, 23]. This is in agreement with the present study, and because of this limited clinical impact, LUS is not considered as a routine part of pretherapeutic evaluations of patients with presumed curable cancers in the stomach, pancreas, or colon. Interestingly, previous studies of smaller study populations found that diagnostic laparoscopy in combination with LUS changed treatment strategy in up to 38 % of the patients [24, 25]. It is noteworthy that both studies are more than 20 years old, and that the technology regarding preoperative imaging like CT has improved since then. That might be one explanation why the clinical impact of LUS was reported that high. A recent systematic review about intraoperative ultrasound and LUS in patients with colorectal cancer also found that LUS detected 13 % additional liver metastases compared to contrast-enhanced CT [18]. This systematic review included few studies on LUS, and no meta-analysis was performed. Another systematic review included more studies regarding LUS, and found that LUS detected between 3 and 13 % additional patients with liver metastases compared to preoperative contrast enhanced CT [20].

One might speculate if the potential impact of LUS would be higher in patients with PM because extraperitoneal malignant disease is probably more likely in these patients compared to patients considered not to have PM. Thus, in a study on the localization of metastases in patients with colorectal cancer, 34 % of the patients with PM also had synchronous liver metastases [26]. Despite this high incidence of liver metastases in patients with PM, LUS had limited clinical impact in our study, which is probably because extraperitoneal metastatic disease had already been identified by CT, MRI, or PET/CT scan and the patient would therefore not be scheduled for PIPAC.

LUS was incomplete in about one third of the cases. This was mainly due to intraabdominal adhesions where access to all parts of the liver or retroperitoneum was not possible. There were no sign of any systematic selection bias towards more aggressive disease in these patients. Thus, the potential inclusion of these patients would not have altered the outcome of the study.

Routine LUS during PIPAC was not fully implemented until March 2018, which is an obvious limitation related to the retrospective study design.

This study only registered the number of patients with “positive” findings at LUS i.e., additional extraperitoneal metastatic disease, which would be a contraindication to PIPAC. Thus, our study did not correlate the findings at LUS with the findings at 14 days follow up CT after PIPAC 3. The sensitivity of LUS can therefore not be calculated, but this was not the aim of the study.

The present study showed that limited time was spent on LUS, and this might be another explanation why LUS had a limited clinical impact. A similar time consumption for LUS was observed during a study on robotic-assisted surgery for primary colorectal cancer [27], and no liver metastasis was detected by LUS in this study either.

## Conclusions

Laparoscopic ultrasound may be safely performed, but it has limited clinical impact and cannot be recommended as a routine procedure in patients scheduled for PIPAC.
